# Impact of Fibromyalgia in the Hippocampal Subfields Volumes of Women—An MRI Study

**DOI:** 10.3390/ijerph18041549

**Published:** 2021-02-06

**Authors:** Juan Luis Leon-Llamas, Santos Villafaina, Alvaro Murillo-Garcia, Narcis Gusi

**Affiliations:** 1 Physical Activity and Quality of Life Research Group (AFYCAV), Faculty of Sport Sciences, University of Extremadura, Av. De Universidad s/n, 10003 Caceres, Spain; leonllamas@unex.es (J.L.L.-L.); svillafaina@unex.es (S.V.); ngusi@unex.es (N.G.); 2 International Institute for Innovation in Aging, University of Extremadura, 10003 Caceres, Spain

**Keywords:** chronic pain, hippocampus, brain, cognitive impairment

## Abstract

Patients with fibromyalgia (FM) show widespread pain associated with other symptoms such as cognitive problems, depression, and anxiety among others associated with alterations in the central nervous system. The hippocampal subfields had differences in function, histology, and connectivity with other brain regions, and are altered in different diseases. This study evaluates the volumetric differences between patients with FM compared with a healthy control group. A total of 49 women with, and 43 healthy women completed this study. T1-weighted MRI was used to assess brain volume, and FreeSurfer software was used to segment the hippocampal subfields. Women with FM had a significant reduction in most of the hippocampal subfields. The regression equation models were obtained to predict the volume of specific subfields of the right and left hippocampus. These findings provide that women with FM have lower hippocampal subfields volumes compared with healthy women. Besides, regression models show that different covariates, such as age, cognitive impairment, or depression, are related to specific subfields.

## 1. Introduction

Fibromyalgia (FM) is a chronic, persistent, and diffuse disease that fluctuates in intensity, characterized by widespread pain and tenderness, accompanied by other numerous symptoms like cognitive problems, depression, anxiety, non-recovery sleep, fatigue, stiffness, poor physical fitness, and mobility or balance problems [1,2,3]. These symptoms lead to a reduced quality of life [4,5] and difficulties in carrying out activities of daily living [6,7]. The prevalence of FM in the general population is between 0.2 and 6.6% [8], occurring mostly in women over 50 years old [9].

Previous research has reported central nervous system (CNS) abnormalities in people with FM [10], including functional, metabolic, and structural alterations in brain regions involved in pain processing [11,12,13,14,15,16]. Besides, alterations in the immune system, sleep patterns, fatigue, and moods contributed to pain and dysfunction in people with FM. This alteration can lead to allodynia (increased pain from a normally non-painful stimulus) and hyperalgesia (increased response to painful stimuli) [17] associated with amplification of peripheral and central sensory signals involved in pain perception [18], affecting the functionality and structure of CNS. In this regard, people with FM show volume reductions in the gray matter of numerous brain regions involved in the “pain matrix” like hippocampus [13,19], prefrontal cortex [20], amygdala [20], anterior cingulate [20,21,22], midcingulate, and midinsula [21] cortices. 

The hippocampus is one of the most studied brain structures that play an important role in numerous processes, including memory, navigation, cognition, moods, stress, and pain [23,24,25,26] related to FM symptomatology. A previous review focused on FM showed alterations in the volume and metabolite levels of the hippocampus [12]. This region is composed of a series of sub-regions such as the subiculum, the dentate gyrus and cornus ammonis [27] among others. Advances in neuroimaging have allowed their study [28], showing that each sub-region had differences in function, histology, connectivity with other brain regions, and vulnerability to disease [29,30,31]. Furthermore, previous studies showed that these sub-regions are altered in chronic pain [32], cognitive disorders [33,34,35], psychiatric disorders [36,37], and adult lifespan [38] being able to become possible biomarkers. 

To our knowledge, no studies have been evaluated the hippocampal subfields volume in people with FM compared with a healthy control group. It seems more relevant to consider the subfields of the hippocampus, rather than just evaluating the whole hippocampus, to more deeply understand the neurobiology of FM. Thereby, this study aims to evaluate the changes in the volume of the hippocampal subfields in women with FM compared with a healthy control group by magnetic resonance imaging (MRI) analysis.

## 2. Materials and Methods

### 2.1. Participants

A total of 50 women with FM from a local association (AFIBROEX) and 43 healthy women from the Open Access Series of Imaging Studies (OASIS-3) participated in this study and, therefore, were divided into two groups: (1) FM group and (2) healthy control group (HC). FM participants met the following inclusion criteria: (a) female and ages between 30 and 75 years, (b) diagnosed with FM by a rheumatologist according to the 2010 American College of Rheumatology criteria [1], (c) able to communicate, and (d) have read and signed the written informed consent. On the other hand, the FM participants were excluded if they: (a) were pregnant, (b) had any cerebral injury (traumatic brain disease, cerebral stroke, brain tumor, or any other diagnosed pathology), and (c) illegible MRI sequences were obtained.

The HC group was obtained by using the OASIS-3 data set. OASIS-3 is a compilation of MRI and PET imaging and related clinical data from 1098 participants, of which 605 are cognitively normal adults, and 493 have various cognitive decline stages. Since different studies have shown brain structural changes associated with cognitive impairment, the sample was filtered to obtain healthy subjects of similar age and gender to the FM group to homogenize the sample. The following inclusion criteria were established: (a) be female and ages between 42 and 60 years, (b) have completed the Mini-Mental State Examination score between 28 to 30, (c) have completed the Geriatric Depression Scale score between 0 to 5, (d) height between 152 to 178 cm, (e) weight between 40 to 120 kg, and (f) have been assessed by a T1w MRI scan with a 3.0 Tesla scanner.

The study was approved by the Research Ethics Committee of the University of Extremadura (approval reference: 62/2017). All participants gave their written informed consent following the updated Declaration of Helsinki.

### 2.2. Image Acquisition

T1-weighted images were obtained using a 3.0 Tesla scanner. For the FM group, a system equipped with an 8-channel head coil (Achieva 3.0 TX, Philips Medical Systems, Best, The Netherlands) was used to obtain the structural images. T1-weighted images were acquired using a 3D T1-weighted Turbo Field Echo (T1-w TFE) sequence with the following parameters: repetition time (TR) of 11.51 ms; echo time (TE) of 2.8 ms; 288 × 288 matrix size; 0.9 mm slice thickness; 10° flip angle; 1 number of averages. For the HC group, a system equipped with a 16-channel head coil (Siemens TIM Trio or BioGraph mMR PET-MR, Erlangen, Germany) was used. The MP-RAGE protocol of TIM Trio scanner used the following parameters: TR/TE = 2400/3.16 ms, ±176 axial slices without slice gap, and 1.0 mm nominal isotropic resolution (FOV = 256 × 256 mm). The MP-RAGE sequence of BioGraph mMR PET-MR scanner used the following parameters: TR/TE = 2300/2.95 ms, ±176 axial slices without slice gap, and 1.2 mm nominal isotropic resolution (FOV = 256 × 256 mm).

### 2.3. Image Processing

**Figure 1:**
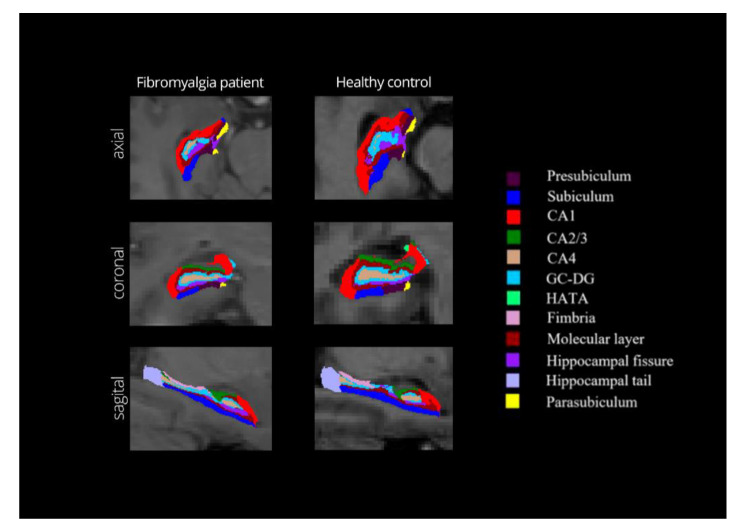
Subfields of the right hippocampus obtained from the FreeSurfer viewer in axial, coronal, and sagittal planes. One participant with fibromyalgia and one control participant with similar characteristics and age 59 years are shown.

All T1-weighted images were processed using the FreeSurfer software [39] 6.0 version (Laboratory for Computational Neuroimaging, Athinoula A. Martinos Center for Biomedical Imaging, Charlestown, MA, USA; http://surfer.nmr.mgh.harvard.edu). The command *recon-all* (http://surfer.nmr.mgh.harvard.edu/fswiki/recon-all) was used for automated segmentation of the T1-weighted images on a MacBook Pro (Version OS X 10.14, 8GB, 2.30 GHz, Intel Core i5). The following steps were followed for the pre-processing of the image data: (a) head motion correction and averaging [40]; (b) removal of non-brain tissue by a hybrid watershed/surface deformation algorithm [41]; (c) automated Talairach space transformation [42]; (d) segmentation of the subcortical and cortical structures using a probabilistic brain atlas [43]; (e) intensity normalization [44]; (f) tessellation of the gray matter and white matter boundary [45]; (g) topology correction; and (h) surface deformation following intensity gradients to reconstruction [43]. Finally, the Iglesias et al. [28] validated method was used to obtain the hippocampal subfields segmentation and volumetric measurements of participants. This procedure uses a probabilistic atlas of the hippocampus combining ex vivo and in vivo MRI data through Bayesian inference, which can automatically segment the hippocampal regions in vivo. For a detailed overview, see Iglesias et al. [28]. The automated segmentation allows to obtain twelve subfields separated by right and left hemisphere: hippocampal tail, parasubiculum, presubiculum, subiculum, cornu ammonis 1 (CA1), cornu ammonis 2/3 (CA2/3), cornu ammonis 4 (CA4), hippocampus-amygdala transition area (HATA), granule cell layer of dentate gyrus (GC-DG), molecular layer, fimbria, and hippocampal fissure. It also includes the total volume of the left and right hippocampus. Figure 1 shows the different hippocampal subfields of a healthy participant and a participant with FM.

### 2.4. Outcome Measurements

Height, weight, age, general depressive state, and cognitive impairment were collected in both groups (FM and HC). HC group outcomes were obtained from the OASIS-3 database. The FM group was assessed through a standardized interview. The following instruments were used for this purpose:

The 15-items Geriatric Depression Scale (GDS) [46] is a questionnaire that allows assessing symptoms of depression. Items are marked with a simple yes/no format. To consider the possible existence of depression symptoms, the cut-off point is set at 5 or higher. For the FM group, the Spanish version was administrated [47].

The Mini-Mental State Examination (MMSE) [48] is a wide dementia screening tool used in clinical practice and different types of studies. This is a written test with a maximum score of 30 points, with lower scores indicating more severe cognitive impairments. The cut-off point is usually set at 24 for “normal” cognitive function. However, it has several limitations depending on the education and age of the participants that must be taken into account [49]. The Spanish version was used with the 30-point version to establish international comparisons [50].

Furthermore, the Fibromyalgia Impact Questionnaire (FIQ) [51] in the Spanish version [52] was used to assess the disease impact. This instrument has 10 items with 3 domains: function, overall impact, and symptoms.

### 2.5. Statistical Analysis

Statistical analysis was carried out using Statistical Package for Social Sciences software (SPSS, version 24.0, IBM Corp, Armonk, New York, NY, USA). Parametric and non-parametric tests were conducted based on the results of the Shapiro-Wilk test.

Mann Whitney *U* test was conducted to examine differences between groups (FM and HC) in depression levels and cognitive functions through the GDS-15 and MMSE questionnaires.

To predict the value of the hippocampal subfields and the whole hippocampus, and determine if there were volumetric differences between the groups the multiple linear regression was used. This analysis also allows determining the relative contribution of independent or predictor variables to the total variance explained in the same direction. Before carrying out the multiple linear regression analysis, it was necessary to comply with the necessary assumptions to obtain valid results. Among these assumptions, the dependent variable needed to be measured on a continuous scale, there are two or more independent variables, there is independence of residues, there is a linear relationship between the dependent variable and each of the independent variables, there is homoscedasticity in the data, and they do not show multicollinearity. Finally, the residuals (errors) follow a normal distribution. The independent variables selected were age, estimated intracranial volume (eTIV), group, GDS-15 score, and MMSE score [33,38].

ANOVA test was performed to test whether the regression model had a good fit for the data. The values of the statistics obtained in the multiple linear regression model were R^2^ or coefficient of determination representing the proportion of variance in the dependent variable that can be explained by the independent variables. The unstandardized coefficients or B indicate how much the dependent variable varies with an independent variable when all other independent variables are held constant.

The level of statistical significance was set at 0.05.

## 3. Results

The demographic characteristics of the FM and HC groups are shown in Table 1. The Mann-Whitney *U* test showed that HC group obtained significantly better results than the FM group in the GDS-15 and MMSE scores (see Table 1). Moreover, FM participants had a moderate effect on the impact of the FM [53]. 

One subject from the FM group was eliminated from the study because an illegible MRI sequence was obtained. Table 2 shows the statistical results of the multiple linear regression of the hippocampal subfields and hippocampal volumes of the FM and HC groups. ANOVA test showed that all models were valid except in the left hippocampal fissure, CA1 bilateral, left fimbria, and HATA bilateral subfields that could not be studied because they did not meet the necessary assumptions to carry out the analysis. 

Multiple regressions to predict the volume of the hippocampal subfields that obtained statistical significance in the independent variables were as follows:

For the right hippocampal subfields, it was found that the variables age, etiv, group, and MMSE predict the LH volume from the equation:

*Predicted ML volume* = 99.63 − (1.50 × *age*) + (*0.00* × *eTIV*) + (7.13 × *MMSE*) + (26.21 × *group*).

The variables eTIV, group and, MMSE predict the CA3 volume, from the equation:

*Predicted CA3* = −114.59 + (0.00 × *eTIV*) + (5.43 × *MMSE*) + (12.42 × *group*)

The age, eTIV, and group predict the CG volume from the equation:

*Predicted GCDG* = 129.11 − (0.84 × *age*) + (0.00 × *eTIV*) + (18.51 × *group*)

For the subfields of the left hippocampus, the variables *age*, *eTIV*, and *group* predicted the volume in the tail, presubiculum, and parasubiculum, from the equations:

*Predicted Tail* = 152.11 − (1.52 × *age*) + (0.00 × *eTIV*) + (110.41 × *group*).

*Predicted presubiculum* = 159.66 − (1.17 × *age*) + (0.00 × *eTIV*) + (23.26 × *group*).

*Predicted Parasubiculum* = 17.20 − (0.31 × *age*) + (0.00 × *eTIV*) + (13.47 × *group*).

As for predicting the volume of the whole hippocampus, the variables predicting volume are age, eTIV, and group for both the left and the right, with the equation *Predicted left hippocampus* = 1737.96 − (10.43 × *age*) + (0.00 × *eTIV*) + (266.52 × *group*) for the left and the equation *Predicted right hippocampus* = 1583.24 − (9.93 × *age*) + (0.00 × *eTIV*) + (270.91 × *group*) for the right.

By performing an individualized analysis of the variables, for each year of age, the volume of the structures decreases in almost all the structures, except the right tail, left CA3, CA4 bilateral, subiculum bilateral, and right presubiculum. In the whole hippocampus, there is also a decrease in volume for each year of age of 10.43 mm^3^ in the left and 9.93 mm^3^ in the right. In contrast, the right fissure presents a volume increase of 0.86 mm^3^ for each year of age.

When visualizing the variable of participation in the HC or FM group, in almost all the subfields, healthy women present a greater volume than women with FM. However, the HC group has 31.96 mm^3^ less than the FM group in the fimbria.

As for the score obtained in MMSE, for each point obtained in MMSE, the volume of right CA3 increases by 5.43 mm^3^.

There are no volumetric changes in the subfields of the hippocampus based on eTIV and GDS scores.

The differences and regression models in the left hippocampal fissure, left and right CA1, left fimbria, and left and right HATA could not be analyzed because the assumptions for conducting the multiple linear regression analysis were not met.

## 4. Discussion

This study aimed to evaluate the volumetric differences in the hippocampal subfields and the whole hippocampus in healthy women and women with FM controlling for age, eTIV, depression, and cognitive impairment. Besides, regression equation models were obtained to predict the volume of the hippocampal subfields and the whole hippocampus. This is the first study that analyzes the hippocampal subfields in women with FM compared to a healthy control group. 

Our results indicated that all regression models were valid. Moreover, the hippocampal subfields and the whole hippocampus had significantly lower volumes in FM than healthy controls except in the right fissure of the hippocampus, where no significant differences were achieved. Considering the whole hippocampus, our results are in line with previous research in FM that also found volumetric reductions in the gray matter of the whole hippocampus [12,13,19]. This has also been suggested in EEG studies through an altered theta, showing that women with FM with more years suffering from symptoms exhibited greater theta power spectrum [54,55]. This is relevant since theta power spectrum is related to higher cognitive functions, synaptic plasticity, and atrophy of the hippocampus [56].

It is now known that reductions in the volume of the hippocampus begin to occur from mid-adulthood. From this point, progressive atrophy of the hippocampus begins to be found as age increases [57]. In this sense, our findings are consistent with the literature since a decrease in volume has been observed in the left and right hippocampus with increasing age. Similarly, these reductions have been detected in most subfields except right tail, left CA3, CA4 bilateral, subiculum bilateral, and right presubiculum. These findings are related to a previous study that has reported a reduction of these subfields with age [35] being the CA subfield the most affected by age. In our study, age-induced decreases in this subfield have not been found. This could be due to the age of participants which was relatively low HC 53.37 (4.47) and FM 54.18 (10.12). Since there is controversy due to the methodological variability among the studies [35], further research is needed to confirm these findings.

The incidence of depression and cognitive impairment in FM is known [58,59], and findings have been reported confirming that patients with major depressive disorder [60] and Alzheimer’s disease (AD) [33] have a smaller hippocampus. However, there is still a non-homogeneous pattern of atrophy in this disease. Thus, our results were controlled for the effect of cognitive impairment and depression, introducing these outcomes as covariates in the statistical analyses. Therefore, findings suggest that higher MMSE scores are associated with volume increases in the right CA3. However, no significant differences were found between GDS scores and hippocampal subfield volumes. In this regard, McCrae et al. [13] found differences in depression comparing HC females and FM females, but no volumetric differences were found in the hippocampus controlling for depression. A previous review of the hippocampal subfields in major depressive disorder [34] reported that volume reductions occurred mainly in the CA and GCDG subfields. Our results did not show volumetric changes in these subfields, which could hypothetically be explained by the higher levels of depression symptoms in major depressive disorder than in FM and the age of participants. In this regard, a previous study reported the most pronounced depression-related alterations of the hippocampus in older adults than young people due to the cumulative effect of depression [61] and age-related atrophy of the hippocampus [62]. 

When comparing our results with the results obtained by Zhao et al. [33], in which the hippocampal subfields were compared in different groups of patients, including AD patients, normal controls, amnestic mild cognitive impairment patients, and subjective cognitive decline patients, we can observe that the volumes obtained in our population of women with FM are lower than the subjective cognitive decline group and higher than the amnestic mild cognitive impairment group as well as the AD group. This is relevant since, among the symptoms of FM, memory and concentration problems known in the literature as “fibrofog” are recurrent and are considered a clinically important aspect of FM [63]. However, our study found no specific effect caused by cognitive impairment in the hippocampal subfields. This effect was probably not found, as no specific neuropsychological tests were carried out in our research to control for cognitive performance in different domains that have been altered [64]. Therefore, future research should study the possible volumetric changes in hippocampal subfields associated with different cognitive domains in FM through different neuropsychological tests. It is known that depression, anxiety, pain, or sleeping disturbances can negatively affect cognitive symptoms. However, they do not entirely explain all the cognitive symptoms of FM [65]. Nevertheless, morphological investigations show decreases of grey material in FM in regions related to cognitive components [66,67]. As we expected, the values obtained in depression and cognitive impairment were worse in the FM group than the HC group, being in line with previous studies that obtained similar results [13,59,68,69]. However, more studies are needed to know the mechanisms involved in their origin.

Regarding the volumetric results obtained in the HC group in some structures are lower when compared to the subjective cognitive decline patient group. These differences could be due to the segmentation methods used. 

While the findings of this study are promising, some limitations must be taken into account. The differences in the left hippocampal fissure, left and right CA1, left fimbria, and left and right HATA could not be analyzed because the assumptions for conducting the multiple linear regression analysis were not met. We only evaluated a sample of women, so these results cannot be generalized to men with FM. The MRI scanners’ possible effects could not be assessed since the sample was not randomized to be measured on the different devices. Furthermore, the pharmacological history of subjects was not an inclusion criterion in the present study. On the other hand, this study was based on cross-sectional data; future research is requested to conduct longitudinal follow-up studies of the same cohort to determine the evolution of these structures to identify possible biomarkers in FM. Finally, we know that the segmentation method is based on an atlas developed from elderly subjects [28], which may present slight hippocampal atrophy. However, this method has shown test–retest reliability in estimating hippocampal volumes and hippocampal subfields [70]. Future research should also be conducted to monitor the stress level and the levels of glucocorticoids generated in the hippocampus. These elements seem to negatively affect the neuronal plasticity of the hippocampus and may influence the reduction of the volume of this structure [12]. In the same way, it would be interesting to consider whether the subjects are medicated to establish possible relationships between decreases or increases in volume in the subfields of the hippocampus [34].

## 5. Conclusions

To our knowledge, this is the first study to analyze volume differences in the subfields of the hippocampus between healthy controls and women with FM. Our findings showed that women with FM had a significant reduction in most of the hippocampal subfields. Besides, regression models show that different covariates, such as age, cognitive impairment, or depression, are related to specific subfields. 

## Figures and Tables

**Table 1:** Characteristics of the participants.

Variables	HC (*n* = 43)	FM (*n* = 49)	Z	*p*-Value
Age (years)	53.37 (4.47)	54.18 (10.12)	−0.051	0.959
Height (cm)	165.07 (6.30)	159.61 (6.24)	−3.972	<0.001
Weight (Kg)	73.92 (6.30)	71.57 (14.05)	−0.767	0.443
GDS-15	2.55 (1.71)	6.88 (4.07)	−5.338	<0.001
MMSE	29.51 (0.63)	28.47 (1.42)	−3.809	<0.001
FIQ-100	-	58.05 (17.85)		
Years with FM Diagnosed	-	10.80 (6.33)		
Years with FM Symptoms	-	19.38 (12.70)		

GDS: Geriatric Depression Scale; MMSE: Mini Mental State Examination; FIQ: Fibromyalgia. Impact Questionnaire; *n*: number of participants; FM: Fibromyalgia; HC: Healthy Control.

**Table 2:** Volume measures and multiple linear regression analysis of the hippocampal subfields and the whole hippocampus.

				ANOVA		Multiple Linear Regression						
Structures		FM Mean (SD)	HC Mean (SD)	F	*p*	Covariates	R^2^	Constant	B	β	t	*p*
Tail	L	402.28 (51.76)	499.09 (67.58)	26.93	<0.001	Age	0.48	152.11	−1.52	−0.16	−2.02	0.046
						eTIV			0.00	0.28	3.33	0.001
						Group			110.41	0.72	8.72	<0.001
	R	397.11 (60.20)	526.63 (76.40)	38.23	<0.001	Age	0.57	4.53	−1.23	−0.10	−1.46	0.148
						eTIV			0.00	0.31	4.15	<0.001
						Group			149.73	0.79	10.57	<0.001
Fissure	L _a_	145.84 (25.58)	147.71 (24.56)									
	R	156.69 (25.52)	143.50 (24.69)	5.79	<0.001	Age	0.17	68.00	0.86	0.27	2.70	0.008
						eTIV			0.00	0.19	1.76	0.081
						Group			−8.65	−0.17	−1.60	0.113
ML	L	513.41 (47.02)	530.38 (59.16)	8.65	<0.001	Age	0.29	99.63	−1.50	−0.22	−2.40	0.019
						eTIV			0.00	0.47	4.85	<0.001
						Group			26.21	0.24	2.30	0.024
						MMSE			7.13	0.17	1.60	0.112
	R	526.55 (51.20)	539.68 (62.52)	11.27	<0.001	Age	0.28	316.88	−2.04	−0.29	−3.11	0.003
						eTIV			0.00	0.49	5.00	<0.001
						Group			29.78	0.26	2.69	0.009
CA1	L _a_	597.81 (68.26)	586.52 (64.12)									
	R _a_	615.80 (67.30)	605.79 (68.15)									
CA3	L	172.80 (21.51)	193.25 (30.17)	11.30	<0.001	Age	0.28	60.52	−0.52	−0.15	−1.62	0.109
						eTIV			0.00	0.39	4.01	<0.001
						Group			27.21	0.49	5.06	<0.001
	R	196.23 (21.99)	205.73 (32.92)	11.56	<0.001	eTIV	0.29	−114.59	0.00	0.50	5.14	<0.001
						Grupo			12.42	0.22	2.11	0.038
						MMSE			5.43	0.24	2.40	0.019
CA4	L	216.59 (22.12)	239.00 (29.54)	13.48	<0.001	Age	0.39	−13.78	−0.57	−0.16	−1.89	0.062
						eTIV			0.00	0.47	5.22	<0.001
						Group			26.70	0.48	4.84	<0.001
						MMSE			3.42	0.15	1.59	0.116
	R	233.31 (22.21)	243.08 (30.98)	12.13	<0.001	Age	0.30	94.36	−0.55	−0.16	−1.81	0.074
						eTIV			0.00	0.54	5.58	<0.001
						Group			18.51	0.30	3.15	0.002
Subiculum	L	395.72 (40.41)	402.66 (49.56)	6.68	<0.001	Age	0.19	219.47	−0.90	−0.16	−1.64	0.104
						eTIV			0.00	0.43	4.19	<0.001
						Group			20.15	0.22	2.17	0.033
	R	394.96 (38.44)	405.08 (47.62)	5.39	0.002	Age	0.16	245.46	−0.83	−0.15	−1.53	0.129
						eTIV			0.00	0.38	3.61	0.001
						Group			21.24	0.25	2.34	0.022
Presubiculum	L	271.72 (32.29)	285.32 (34.14)	10.47	<0.001	Age	0.27	159.66	−1.17	−0.28	−2.99	0.004
						eTIV			0.00	0.43	4.37	<0.001
						Group			23.26	0.35	3.52	0.001
	R	257.34 (30.47)	276.78 (32.49)	8.05	<0.001	eTIV	0.22	91.36	0.00	0.34	3.37	0.001
						Group			35.46	0.54	4.45	<0.001
						GDS			1.82	0.21	1.80	0.076
GC-DG	L	256.51 (25.87)	277.44 (33.87)	11.52	<0.001	Age	0.35	22.67	−0.89	−0.23	−2.54	0.013
						eTIV			0.00	0.46	4.90	<0.001
						Group			25.50	0.41	4.00	<0.001
						MMSE			3.42	0.15	1.59	0.116
	R	273.89 (25.30)	282.61 (35.25)	12.36	<0.001	Age	0.30	129.11	−0.84	−0.22	−2.40	0.018
						eTIV			0.00	0.54	5.58	<0.001
						Group			18.51	0.30	3.15	0.002
Fimbria	L _a_	102.73 (18.72)	75.33 (16.50)			Age						
						Group						
	R	107.37 (18.99)	73.42 (14.38)	48.64	<0.001	eTIV	0.53	102.12	0.00	0.15	1.91	0.060
						Group			−31.96	−0.66	−8.47	<0.001
Parasubiculum	L	45.91 (8.20)	57.24 (11.65)	16.16	<0.001	Age	0.36	17.20	−0.31	−0.22	−2.49	0.015
						eTIV			0.00	0.27	2.88	0.005
						Group			13.47	0.59	6.44	<0.001
	R	45.97 (8.63)	56.20 (11.02)	13.88	<0.001	Age	0.22	46.85	−0.20	−0.15	−1.57	0.119
						Group			10.07	0.46	4.94	<0.001
HATA	L _a_	58.09 (7.48)	56.49 (9.80)									
	R _a_	61.12 (7.70)	58.52 (8.98)									
Hippocampus	L	3033.93 (263.48)	3202.74 (334.79)	14.84	<0.001	Age	0.34	1737.96	−10.43	−0.27	−3.05	0.003
						eTIV			0.00	0.49	5.25	<0.001
						Group			266.52	0.43	4.62	<0.001
	R	3109.64 (286.93)	3273.53 (346.07)	15.19	<0.001	Age	0.35	1583.24	−9.93	−0.24	−2.77	0.007
						eTIV			0.00	0.53	5.66	<0.001
						Group			270.91	0.42	4.48	<0.001

Abbreviations: FM, fibromyalgia; HC, healthy control; SD, standard deviation; ML, molecular layer; CA, cornus ammonis; GC-DG, granule cell-dentate gyrus; HATA, hippocampus—amygdala-transition-area; eTIV, estimated total intracranial volume; MMSE, mini-mental state examination; GDS, geriatric depression scale; R^2^, coefficient of determination; B, unstandardized coefficient; β, standardized coefficient; *p*, *p*-value. _a_ The assumptions for performing the multiple linear regression are not met, FM = 1; HC = 2.

## Data Availability

The data of the healthy control group presented in this study are openly available in https://www.oasis-brains.org/.
